# Effectiveness of the Relaxation Response-Based Group Intervention for Treating Depressed Chinese American Immigrants: A Pilot Study

**DOI:** 10.3390/ijerph110909186

**Published:** 2014-09-05

**Authors:** Albert Yeung, Lauren E. Slipp, Halsey Niles, Jolene Jacquart, Choi-Ling Chow, Maurizio Fava, John W. Denninger, Herbert Benson, Gregory L. Fricchione

**Affiliations:** 1 Benson-Henry Institute for Mind Body Medicine, Massachusetts General Hospital, 151 Merrimac St., Boston, MA 02114, USA; E-Mails: lslipp@partners.org (L.E.S.); halsey.niles@gmail.com (H.N.); jjacquart@mgh.harvard.edu (J.J.); jdenninger@mgh.harvard.edu (J.W.D.); hbenson@mgh.harvard.edu (H.B.); gfricchione@mgh.harvard.edu (G.L.F.); 2 South Cove Community Health Center, 885 Washington St., Boston, MA 02111, USA;E-Mail: ochow@scchc.org; 3 Depression Clinical and Research Program, Massachusetts General Hospital, 1 Bowdoin Square, Boston, MA 02114, USA; E-Mail: mfava@mgh.harvard.edu; 4 Department of Psychiatry, Harvard Medical School, 25 Shattuck St., Boston, MA 02115, USA; 5 Department of Medicine, Harvard Medical School, 25 Shattuck St., Boston, MA 02115, USA

**Keywords:** depression, Chinese, relaxation response, mind-body, group intervention

## Abstract

*Background*: This study examined the feasibility, safety and efficacy of an 8-week Relaxation Response (RR)-based group. *Methods*: Twenty-two depressed Chinese American immigrants were recruited. Outcomes measures were response and remission rates, the Hamilton Rating Scale for Depression, Clinical Global Impressions Scale, Quality of Life Enjoyment and Satisfaction Questionnaire, and the Multidimensional Scale of Perceived Social Support Scale. *Results:* Participants (N = 22) were 82% female, mean age was 53 (±12). After intervention, completers (N = 15) showed a 40% response rate and a 27% remission rate, and statistically significant improvement in most outcome measures. *Discussion*: The RR-based group is feasible and safe in treating Chinese American immigrants with depression.

## 1. Introduction

### 1.1. Depression and Health Disparities among Chinese Americans

According to the World Health Organization (WHO Depression), depression was the leading cause of disability, as measured by years lived with disability, and the fourth leading contributor to the global burden of disease measured in “disability adjusted life years” in 2000 [1]. Patients with untreated depression suffer higher medical costs, worse health outcomes, and lower quality of life than those whose depression is treated [2]. There are tremendous disparities in the treatment of depression in the U.S. and Asian Americans were reported to have the lowest utilization of mental health services [3]. Ethnic minorities face both practical and cultural barriers to mental health care. They frequently lack the resources to seek help, suffer from language barriers, and view psychiatric illnesses as stigmatizing [3]. These lead to under-recognition and under-treatment of depression among racial minorities, including among Asian immigrants [4,5,6,7,8,9,10]. The Surgeon General’s Report considered correcting these disparities a top priority, and suggested offering minority-centered services and developing culturally-competent care to address the specific needs of minority patients [3].

### 1.2. Promise of Mind-Body Interventions

Mind-body interventions are practices designed to facilitate the mind’s capacity to affect health are now common therapies in the U.S., with meditation, imagery, and yoga being the most frequently used techniques [11]. National Survey data from 2004 indicate that almost 1 in 5 people in the U.S. used at least one mind-body therapy that year [11]. Out-of-pocket expenditures for mind-body therapies totaled more than $5 billion in 2007 [12]. Mind-body techniques like meditation, imagery, and yoga show great potential for becoming widely integrated into the prevention and rehabilitation of a number of medical conditions, including mental illnesses. One of the benefits of using such techniques is that they appear to be safe [13], even for the elderly and the physically frail. Clinical and community-based studies have reported high adherence and enjoyment [14]. Moreover, most mind-body therapies appear to be relatively cost-effective, requiring no special equipment or facilities [15]. Mind-body interventions may be more acceptable to Chinese as they are generally viewed as well-being practices and are not associated with stigmas against psychiatric disorders. 

There is a considerable body of clinical research, including work from our group, providing evidence on the benefits of mind-body intervention for a variety of health issues including cardiovascular and neuromuscular conditions [16,17,18,19,20,21,22], as well as for psychological conditions including depression [23,24,25,26,27,28]. In depression-specific research, a growing body of research points to the efficacy of Mindfulness Based Cognitive Therapy (MBCT), a popular mind-body group intervention, for treating major depressive disorder (MDD). MBCT is an 8-week, structured, mind-body group program which combines the elements of cognitive therapy with mindfulness meditation training. MBCT has been demonstrated to reduce the risk of relapse in patients with three or more prior depressive episodes [29,30,31], and more recent preliminary studies suggest that it may be used as an active treatment for current depression [32,33,34,35,36]. 

### 1.3. The Relaxation Response-Based Group Intervention

The cornerstone of the Relaxation Response-based Group Intervention is the Relaxation Response (RR), thought to be central to many mind-body techniques, which is described as a “wakeful hypometabolic state” [37] found to be effective in counteracting maladaptive responses to stress responses described by Cannon [38] as the “fight-or-flight” response. The RR is a physiological state characterized by decreased arousal of the sympathetic nervous system [37], and can be elicited using many mind-body techniques, including breath awareness, self-hypnosis, meditation, and yoga. The physiological, psychological, functional, structural, and genomic effects of eliciting the RR have been reported in earlier studies [39,40,41,42,43,44,45,46,47,48,49,50,51,52,53,54,55]. For over 25 years, the Benson-Henry Institute for Mind Body Medicine (BHI) at the Massachusetts General Hospital (MGH) has been offering an RR-based group intervention. It was designed to promote resiliency by reducing the harmful effects of stress through elicitation of the RR and skills training in cultivating positive beliefs, nutrition, exercise, recuperative sleep, social support and coping [56]. In the RR-based Group discussed here, patients are taught to use muscle relaxation, breathing techniques, focused awareness and open monitoring meditation, yoga, and imagery to elicit the RR.

In 2010, the BHI RR-based group intervention was re-designed from its former 12-session structure to an 8-session group format (content described in Table 1). More recently, The RR-based group intervention evolved into the Relaxation Response Resiliency (3RP) Program [57], which covers three core target areas: (1) elicitation of the RR; (2) stress appraisal and coping; and (3) growth enhancement. The group intervention has been shown to reduce medical symptoms of patients who suffer from chronic medical illnesses [56], facilitate the elimination of antihypertensive medication among patients with hypertension [58], and lower the risk factors for cardiac events among participants of a cardiac rehabilitation program [59]. However, the participants of these studies were predominantly white, with relatively high education levels and socio-economic status. 

In this study, we examined the safety and efficacy of the RR-based group intervention for treating underserved Chinese American immigrants diagnosed with MDD. We hypothesize that an RR-based group intervention is both feasible and effective in the treatment of MDD in this population.

## 2. Experimental Section 

### 2.1. Participants

Twenty-two Chinese Americans were recruited from the primary care clinics at South Cove Community Health Center (South Cove) between October 2010 and November 2011. South Cove is a federally-funded community health center in Boston that predominantly serves Chinese Americans. In 2011, South Cove served more than 25,000 patients, the majority of whom (>92%) were Chinese immigrants. All participants were required to be fluent Chinese speakers or at least understand Cantonese Chinese, as the group was conducted in Cantonese Chinese. All subjects gave their informed consent for inclusion before they participated in the study. The study was conducted in accordance with the Declaration of Helsinki, and the protocol was approved by the Ethics Committee of Massachusetts General Hospital (Project identification code 2009P002557).

**Table 1:** RR-based group intervention chapters and goals.

Chapter	Content
**Chapter 1** Stress and the Relaxation Responses (RR)	Introduce RR elicitation method: breath awareness Describe the relationship between the RR, health, and wellness Identify individual’s sources of stress and coping Introduce “Minis” as a method to reduce tension and anxiety throughout the day
**Chapter 2** Mindfulness and Appreciation	Introduce RR-elicitation method: mindful awareness Use strategy for applying mindful awareness in daily living Learn concepts and strategies for enhancing positivity Identify how positivity can increase resiliency in the long-term
**Chapter 3** Thoughts, Feelings, and Behaviors	Build awareness of physical, emotional, and cognitive expression of stress Introduce cognitive distortion: Review negative beliefs that prevent adaptation Introduce cognitive restructuring: Build adaptive beliefs: problem solving, reframing, and positive expectation
**Chapter 4** Yoga, Exercise, and Movement	Introduce Mind-body benefits of physical activity Introduce Strength Training to build lean body mass Introduce gentle stretching and body awareness Introduce Hatha yoga
**Chapter 5** Using Imagery to Relax and Heal	Create imaginative perspectives to influence new behaviors and to gain insight Introduce new RR-elicitation method: joyful place imagery Use contemplation to deepen positive perspectives through meditation
**Chapter 6** Mindful Eating and Nutrition	Develop balanced and healthful attitudes toward food Pleasure, satiety, and dietary disinhibition Mindful eating Learn label reading and building a diet of desirable nutrients
**Chapter 7** Humor and Optimism/Sleeping Well	Identify Mind-body benefits of laughter and amusement Strategies to bring humor into life Practice using humor to enhance processes of appreciation and acceptance Introduce good sleep practices
**Chapter 8** Goals and Planning	Review mind-body intervention strategies learned Develop a plan for continuing to use program strategies Set goals for the future Ways to prevent relapse

Inclusion criteria included: (1) Self-identification as of Chinese ethnicity, fluent in Mandarin and/or Cantonese, or at least understand Cantonese Chinese; (2) 18–65 years of age; (3) Satisfy Diagnostic and Statistical Manual of Mental Disorders, Fourth Edition (DSM-IV) diagnosis of MDD, as determined by the SCID interview; and (4) Baseline score on the 17-item Hamilton Rating Scale for Depression (HAM-D_17_) ≥12. Exclusion criteria included: (1) Primary psychiatric diagnosis other than MDD; (2) History of psychosis, mania, or severe cluster B personality disorder; (3) Judged by the investigators to have unstable medical conditions; (4) Having current active suicidal or self-injurious potential necessitating immediate treatment, (5) Have had CBT treatment in the past 3 months, and (6) Regular practice of any mind-body technique, including yoga, Tai chi, or Qigong, *etc.* in the past three months. Participants receiving treatment for depression, including antidepressants, conventional psychotherapy, or complementary treatments, were allowed to continue their existing treatments and advised not to make changes during the study.

### 2.2. Participant Enrollment

Recruitment fliers advertising stress management training were placed at South Cove. This is considered a culturally-sensitive way to recruit less acculturated Chinese patients, who fear the stigma of depression and other mental illnesses. In addition, primary care physicians and behavioral health clinicians at South Cove were encouraged to refer patients to this study.

Potential participants were pre-screened over the phone by our bilingual research staff using an IRB-approved protocol. They were then scheduled for a screening visit, where a bilingual investigator obtained informed consent from each participant and conducted interviews. A psychiatrist administered the Chinese Bilingual version of the semi-structured psychiatric interview (CB-SCID-I/P) [60] to assess the diagnosis of MDD and to rule out other disorders, and administered the HAM-D_17_ to determine eligibility. 

### 2.3. Intervention

The RR-based group intervention followed the manualized protocol of the BHI RR-based group intervention, and consisted of 1.5 h group classes held weekly for eight weeks, in a conference room at the health center. The protocol provided a comprehensive introduction to the relationship between the mind and the body. It included training of breathing awareness and awareness of physical and emotional sensations to attain a mindful approach to everyday living. In addition, techniques for problems solving, the emphasis of positivity, the awareness of negative thoughts and use cognitive reconstruction methods, and the practice of imagery were introduced as part of the group intervention (Table 1). Rather than using deliberate efforts to strive for a relaxed sensation, the intervention employed techniques which were non-striving in nature with “non-judgmental” awareness of the present moment. In 2010, the Medical Interpreter Services at MGH translated the protocol into Chinese, which was revised and edited both by a bilingual psychiatrist (AY) and a bilingual social worker (CLC) on our team to reach consensus regarding the accuracy of the translation. 

The RR-based group intervention was led by an instructor (CLC) with over 16 years experience serving Chinese populations, and extended experience with group treatment. At the time of the intervention, the instructor had received over 13 years of mind-body training, including advanced training on delivering the RR-based group intervention from the BHI. The instructor offered reassurance to participants as initial practice of mindfulness could be associated with increased anxiety due to the awareness of upsetting thoughts or feelings, and approaches to manage such emotions were taught in the group. Participants received a CD with guided meditations in Cantonese or Mandarin Chinese, and were encouraged to practice at home at least six times per week and record the frequency and duration of their practice. Classes were conducted in Cantonese.

### 2.4. Outcome Assessments

Outcome measures were assessed at baseline, week 4, and week 8. At each assessment, participants were administered the HAM-D_17_ [61,62,63,64], the Clinical Global Impressions—Severity (CGI-S) and Improvement (CGI-I) [65], the Quality of Life Enjoyment and Satisfaction Questionnaire, Short-Form (Q-LES-Q-SF) [66], and the Multidimensional Scale of Perceived Social Support (MSPSS) [67,68]. 

The HAM-D_17_ is a widely studied instrument for depression, with high reliability and validity [61]. The Chinese version of the HAM-D_17_ has been shown to have adequate reliability and validity in an earlier study [64]. The CGI-S measures the current condition of the patient, as judged by the clinician, on a scale of 1–7 (1 reflecting normal, and 7 reflecting the most severely ill patients); and the CGI-I measures the degree of improvement, as judged by the clinician, since the start of treatment on a scale of 1–7 (1, very much improved; 4, no change; 7, very much worse) [65].

The Q-LES-Q-SF is a self-report measure and each item is rated from 1 (very poor) to 5 (very good). Results are presented as a percentage of the maximum possible total score, with higher scores representing better quality of life. The Chinese version of the Q-LES-Q-SF has been demonstrated to have good reliability and validity among Chinese patients with psychiatric disorders [69]. 

The MSPSS is a self-administered 12-item scale used to assess perceptions of social support from family members, friends and significant others. Items are rated on a 7-point Likert Scale (1, very strongly disagree; 7, very strongly agree), with higher scores indicating greater level of perceived support. Confirmatory factor analysis has consistently reported a 3-factor solution: family (MSPSS-FA), friends (MSPSS-FR) and significant others (MSPSS-SO) [70,71]. Internal consistency of the Chinese version is good [70,72,73].

In addition, participants were asked to fill out the “Beliefs and Expectations of Mind-Body Group Treatment” [18,74], a 4-point Likert Scale (0 = no, 1 = maybe, 2 = yes, 3 = definitely) to report how effective they considered the RR-based group intervention as a treatment for depression.

### 2.5. Feasibility and Safety Measures

There has been no publication to date on the use of a mind-body intervention group on Asian Americans with depression. This study examined the acceptability of the RR-based group intervention to Chinese American patients with depression, and the degree of adherence of recruited patients [75]. At each class, participants were asked to fill in an attendance sheet and an adherence to mind-body practice log to report the frequency and duration of their practice in the past week. At week 4 and week 8, participants were asked to fill out an adverse event log to monitor possible adverse events related to the intervention. All participants had access by phone at all time through the study to the principal investigator who was a licensed psychiatrist.

### 2.6. Data Analyses

All participants were evaluated at week 4 and week 8 assessments, and measurements made at the baseline were compared to week 8 assessments. Participants were classified as completers, those who attended 6 (75%) or more of the eight training sessions, and non-completers of the intervention. The descriptive and clinical characteristics of completers and non-completers were compared using Fisher Exact Tests and the non-parametric Mann-Whitney U Tests (Table 2).

Outcomes analyses were performed using completer analysis, to include participates who had ≥75% attendance. Positive response to treatment was defined as a decrease of 50% or more of a patient’s HAM-D_17_ score [76], and remission was defined as having a score of 7 or less on the HAM-D_17_ at the last assessment. Participants’ HAM-D_17_, CGI-S, CGI-I, Q-LES-Q-SF and MSPSS measurements before and after the intervention were compared using the non-parametric Wilcoxin Signed Ranks Test, in view of the small sample size. Statistical analyses were conducted using SPSS software, version 20.0 ( IBM Corp., Armonk, NY, USA) [77]. For all analyses, significance was set at the 0.05 alpha level. 

**Table 2:** Baseline characteristics of study participants (N = 22).

Characteristics	Completers (N = 15)		Non-Completers (N = 7)		χ2 (df)/Z Score ^@^	*p*
	% (n)	Mean (SD)	% (n)	Mean (SD)		
Age (years)		53 (13)		51 (11)	−0.81	0.42
Gender (female)	80 (12)		86 (6)		0.1 (df = 1)	1.0
Marital Status						
Married	33 (5)		43 (3)		3.8 (df = 3)	0.29
Widowed	13 (2)		43 (3)			
Separated/divorced	47 (7)		14 (1)			
Never married	7 (1)		0 (0)			
Education (years)		10.8 (4.3)		10.1 (3.6)	−0.29	0.8
Employment Status (employed)						
Full time	13 (2)		0 (0)		6.2 (df = 4)	0.19
Part time	27 (4)		29 (2)			
Home maker	53 (8)		29 (2)			
Retired	7 (1)		14 (1)			
Unemployed	0 (0)		29 (2)			
Currently receiving antidepressants	60 (9)		57 (4)		0.016 (df = 1)	0.90
Beliefs in usefulness of Mind-body Group on depression						
Not helpful	0 (0)		0 (0)		4.5 (df = 2)	0.11
May be helpful	40 (6)		86 (6)			
Helpful	27 (4)		14 (1)			
Definitely helpful	33 (5)		0 (0)			
BDI		27.53 (8.94)		30.1 (8.9)	−0.32	0.75
HAMD 17		17.73 (2.55)		22.1 (3.4)	−2.6	0.009 **
CGI-S (Baseline)		3.80 (0.68)		4.9 (0.7)	−2.7	0.006 **
Q-LES-Q Score, total		0.38 (0.12)		0.3 (0.1)	−0.71	0.48
MSPSS-SO (significant other)		12.93 (7.50)		10.4 (7.3)	−0.61	0.54
MSPSS-FA (family)		13.93 (7.21)		15.2 (7.8)	−0.50	0.62
MSPSS-FR (friends)		12.93 (7.06)		10.7 (7.0)	−0.50	0.62

Notes: ^@^Mann-Whitney U Test, ** Statistically significant (*p* < 0.05), The U values are available upon request.

## 3. Results and Discussion

### 3.1. Results

Twenty-two Chinese American immigrants with MDD were enrolled in the study (82% female, mean age 53 ± 12). The average years of school attended was 10 ± 3.3, and only 36% of participants were employed either full-time or part-time. All were immigrants and recipients of government-funded health care programs for individuals with low incomes. Most participants had positive expectations that the RR-based group intervention would help their depression (“not helpful”: 0%, “maybe”: 55%, “yes”: 22.5%, and “definitely”: 22.5%). At baseline, participants had an average HAM-D_17_ score of 19.1, which corresponds to being severely depressed [62]. Seven participants were non-completers; two found a job and became unavailable, another two had family crises, one did not like the lack of parking space around the health center, and two other participants left the study without offering any reasons. 

Fifteen (68%) participants completed 75% or more of the intervention. No adverse events due to the RR-based group intervention were reported. The completers and non-completers share similar demographic characteristics and expectations about the intervention’s efficacy. However, the completers were found to have lower mean HAM-D_17_ and CGI-S at baseline, suggesting completers were less depressed (Table 2).

At the end of the intervention, 64% of all participants found the group helpful, 59% reported that they practiced at least three days each week, 100% were satisfied with the group, 64% reported that they planned to continue to practice eliciting the RR at home, and 64% reported that they would recommend the group to their families and friends.

The intervention resulted in a treatment response rate of 40% both at week 4 and week 8, and a remission rate of 13% at week 4 and 27% at week 8 demonstrating a trend of improvement over time. For changes in continuous measurements at week 8, there were statistically significant improvement in HAM-D_17_ (from 17.73 ± 2.55 to 11.87 ± 4.75, z = −3.07, *p* = 0.002), CGI-S (from 3.80 ± 0.68 to 3.07 ± 0.88, z = −2.5, *p* = 0.019), CGI-I (mean of 2.67 ± 1.05, z = −2.98, *p* = 0.003), Q-LES-Q-SF (from 0.38 ± 0.12 to 0.47 ± 0.13, z = −3.2, *p* = 0.002), and in MSPSS-FA (from 13.93 ± 7.21 to 15.60 ± 8.08, z = −2.78, *p* = 0.006) which may reflect patients’ more favorable assessment of family support, or improved family relationship when patients became less symptomatic. Both the MSPSS-SO and MSPSS-FR showed improvement after the intervention, but the changes were not statistically significant. The intervention outcome results are shown in Table 3.

### 3.2. Discussion

This pilot study provides preliminary information on the potential impact of an RR-based group intervention for treating depressed immigrant Chinese Americans with lower education and acculturation levels. This population historically underutilizes conventional mental health treatment options [4]. If the acceptability and efficacy of an RR-based group intervention in treating immigrant Chinese with MDD can be shown, it has the potential to significantly impact a large number of underserved ethnic minorities with mental health issues. This study demonstrates the feasibility and safety of using an RR-based group intervention as an intervention for Chinese Americans with MDD. During recruitment, many Chinese patients responded favorably to the idea of stress management, which does not carry the stigma associated with treatment of mental disorders. At baseline evaluation, all participants had positive expectations of the intervention for treatment of depression. 68% of participants completed the intervention (defined as ≥75% attendance), demonstrating satisfactory compliance with treatment. Participants reported no adverse events, high levels of satisfaction and many (64%) were willing to continue their mind-body practice at the end of the study. The health center was receptive to having the mind-body group intervention as it was conducted in a format that is very similar to group psychotherapies. We anticipate that such a program should receive similar acceptance in other health centers if they adopt a similar approach. 

**Table 3:** Depression treatment outcomes using completer analysis (N = 15).

Treatment Outcomes	Initial Assessment		Final (Week 8) Assessment			*z* Value *	*p* Value
	Mean	(SD)	% (N)	Mean	(SD)		
Response rate			40 (6)				
Remission rate			27 (4)				
CGI-S	3.80	(0.68)		3.07	(0.88)	z = −2.50	0.019 **
CGI-I (lower scores reflect more improvement)				2.67	(1.05)	z = −2.98	0.003 **
HAM-D_17_	17.73	(2.55)		11.87	(4.75)	z = −3.07	0.002 **
BDI	27.53	(8.94)		19.97	(13.10)	z = −2.47	0.013 **
Q-LES-Q	0.38	(0.12)		0.47	(0.13)	z = −3.12	0.002 **
MSPSS-SO	12.93	(7.50)		16.40	(7.42)	z = −1.81	0.071
MSPSS-FA	13.93	(7.21)		15.60	(8.08)	z = −2.78	0.006 **
MSPSS-FR	12.93	(7.06)		16.67	(7.93)	z = −1.69	0.093

Notes: *Wilcoxon Signed Rank test. The W values are available upon request. ** Statistically significant (*p* < 0.05).

This study provides valuable information regarding intervention feasibility, outcome measurements, and effect size which will facilitate the design of future studies on mind-body group interventions for the treatment of immigrant populations with lower education and socio-economical levels. We restricted outcome analyses to participants who completed at least 75% of the sessions because we hypothesized that patients need to receive adequate exposure to the intervention in order to improve. This approach was supported, in part, by the evidence that the completers of the intervention had better outcomes in their depressive symptoms compared to the non-completers (as shown by HAM-D and CGI-S), despite the similarities in demographic data and treatment histories between completers and non-completers (Table 2).

The RR-based group intervention showed response rate of 40% and remission rate of 27%. Most mind body intervention studies on depression reported continuous measurements and not categorical outcomes using response/remission rates. The findings of this study approximate those from the STAR-D [76], the largest clinical trial performed in the U.S. on the treatment of MDD patients in the primary care setting using antidepressants which reported remission rates of 28% based on HAM-D and 33% based on the Quick Inventory of Depression Symptomatology (QIDS-SR) [78], and a response rate of 47% based on QIDS-SR. Participants showed significant improvement in mean HAM-D_17,_ BDI, CGI-S, CGI-I, Q-LES-Q, and MSPSS-FA values. Our study adds to the growing number of studies in different continents of the world which show the promise of multimodal mind-body group interventions for patients with depression [79,80,81]. We would like to acknowledge the following limitations of this pilot study. First, this is an open label study with a small sample size and no control group in which all participants received the intervention. The lack of a control group made it impossible to have blind assessment of outcomes and potentially led to biases in rating if raters anticipated favorable outcomes. While we found significant improvements in many outcome variables, the absence of a control group makes it impossible to attribute changes specifically to the intervention. Participants who were taking antidepressant medications may have improved because of medication effects. However, most participants who were on an antidepressant had been treated with medications for a long time (mean duration 10 ± 8 months), and were referred to the group because they were resistant to medication treatment. It is possible that participants’ symptomatic improvement was due to the passage of time, which could happen if participants sought help while they were highly symptomatic, as depressive symptom severity may fluctuate and decrease over time. To fully address this challenge, future studies using a randomized control design are needed. Secondly, it is unclear if participants’ improvement in the intervention group was a result of the regular RR elicitation as part of the group intervention, or of the social interaction involved in participating in the study. Future attention-controlled and mechanistic studies might further investigate the differential impact of an RR-based group intervention and of social interaction. Another limitation is the issue of generalizability. As participants in this study were predominantly recent Chinese immigrants, we cannot be sure whether these results would generalize to other populations. Further studies will be needed to examine if mind-body group interventions are effective for treating depression in the mainstream population and in other ethnic minority groups. 

## 4. Conclusions

The BHI RR-based group intervention, an eight-week mind-body intervention, appears to be a feasible and acceptable intervention for immigrant Chinese Americans with MDD. Future studies with larger sample sizes using randomized controls will be needed to provide more rigorous outcomes analyses. 
